# Determining the reliability of the Vitalight CO_2_ Monitor via the assessment of the carbon dioxide profile on city transit buses

**DOI:** 10.1371/journal.pone.0342566

**Published:** 2026-02-11

**Authors:** Courtney E. Lessel, Katie A. Goggins, Alison A. Godwin

**Affiliations:** 1 Centre for Research in Occupational Safety and Health, Laurentian University, Greater Sudbury, Ontario, Canada; 2 Bharti School of Engineering and Computation, Laurentian University, Greater Sudbury, Ontario, Canada; 3 School of Kinesiology and Health Sciences, Laurentian University, Greater Sudbury, Ontario, Canada; Central Queensland University, AUSTRALIA

## Abstract

Public transit vehicles represent small, dynamic, indoor spaces, with conditions that can be favourable for the development of poor air quality. The use of CO_2_ monitors is recommended as a potential strategy for improving ventilation; however, information around how non-experts can use them for personal monitoring is limited. The purpose of this field study was to assess the real-world usability of a low-cost monitor, and to provide general recommendations around personal risk assessment using a monitor in combination with the evaluation of environmental factors. Measurements of CO_2_ and surveying of commute conditions were carried out with two CO_2_ monitors, the Vitalight and the Aranet, during 250 public transit trips between October 17^th^, 2022 and July 18^th^, 2023. Results show that CO_2_ measurements taken by the Vitalight and the Aranet were highly correlated (Time 1 (T1): r = .948; Time 2 (T2): r = .966). Additionally, the Vitalight paralleled the Aranet with respect to its responses to: temperature; relative humidity; occupancy level; seating position; how often the bus doors opened; and whether the windows were open or closed. However, measurements of CO_2_ taken by the Vitalight were significantly lower than those taken by the Aranet (T1: *t*(249) = −22.52, p < .001; T2: *t*(249) = −32.44, p < .001). Based on these results, we make three recommendations to provide guidance to non-experts around personal monitoring for ventilation on transit buses, including use and interpretation of the Vitalight monitor, and environmental conditions to assess, to inform actions to take to improve ventilation.

## Introduction

Indoor air quality (IAQ) is the air quality in and around buildings or structures [1]. Ventilation, a component of IAQ, is the replacement of indoor air with ambient air [2,3], which helps with the dilution of contaminants [4], including: chemical and biological contaminants, and particulate matter [2]. Managing ventilation is key to maintaining good IAQ, and is a control method for reducing the transmission of airborne infections [3,5–8].

Low IAQ has been correlated with negative health effects [2,4]. For example, high concentrations of carbon dioxide (CO_2_) have been linked to headaches, sleepiness, fatigue, and reduced cognitive function [1,4,8]. Additionally, it has been positively correlated with the transmission of viruses, such as COVID-19 [9]. Recent work indicates that CO_2_ concentrations above 800 ppm increase the stability of COVID-19 in the air [10], increasing it’s chance of transmissibility between humans. Therefore, improving ventilation can decrease the concentration of airborne biological contaminants; potentially helping to destabilize them and, ultimately, improve health outcomes.

There are existing difficulties related to real-time monitoring, and direct measurement, of airborne biological contaminants [11]. Therefore, standards for IAQ generally regulate ventilation and filtration, relative to quantities of non-biological contaminants [12] (e.g., American Society of Heating, Refrigerating and Air-Conditioning Engineers (ASHRAE) standard 62.1–2022 [13]). In indoor spaces, most CO_2_ is produced by the occupants, so it is considered representative of the fraction of air that is composed of exhaled breath [14–16]. Based on this assumption, CO_2_ monitoring is used to indicate whether the level of ventilation is sufficient to dilute airborne contaminants [1–3,14,17–19]. It can also provide information around occupancy, which can be used to control ventilation in real-time (e.g., demand-control ventilation) [2,14].

In 2023, largely as an outcome of concerns raised during the COVID-19 pandemic, ASHRAE published standard 241, which outlines the control of infectious aerosols with ventilation, and air filtration and cleaning [20]. Additionally, greater focus has been placed on the use of personal CO_2_ monitors to assess ventilation as a proxy for infection risk in indoor air [10,11]. The average concentration of CO_2_ in ambient air is approximately 400 ppm [1,3,15,17,21], however, in exhaled breath, the concentration of CO_2_ is approximately 40,000 ppm [3,15,16]. Based on equations calculating the fraction of rebreathed air, at CO_2_ concentrations of 600 ppm, ~ 0.50–0.53% of the air is rebreathed, ~ 0.92–1.00% at 800 ppm, ~ 1.32–1.50% at 1000 ppm, ~ 1.58–2.10% at 1200 ppm, and ~2.89–4.20% at 2000ppm [22,23]. Therefore, when ventilation is insufficient, exhaled CO_2_ and airborne contaminants can build up [4,24]. The level of build-up is affected by: occupancy level [3,14,25]; occupant characteristics [17]; duration of time people are in the space; and activities taking place in the space [3,14].

Real-time CO_2_ monitoring can be used to help define high risk times for transmission, where additional control measures should be implemented [7]. Values from real-time monitoring can also be used with modelling to estimate the relative risk of infection from airborne illnesses [9,16]. Moreover, personal monitoring provides area specific information about short-term exposures that individuals can use for personal risk assessment [4]. In general, CO_2_ limits between 800 and 1000 ppm are recommended as indicators of insufficient ventilation [3,14,26,27]. However, during pandemic conditions, CO_2_ limits should be kept as close to outdoor air conditions as possible [28].

Public transit vehicles represent small, dynamic, indoor spaces [21]. These environments can pose a health risk for transit operators, and passengers, as contacts between people facilitate exposures to airborne biological contaminants [29], such as bacteria and viruses. The urban transit system in Canada recorded an average number of 126 million passenger trips per month between May 2023 and April 2024 [30]. According to data from the Canadian Human Activity Pattern Survey (CHAPS) 2, individuals spend approximately five-percent of their day in vehicles, with urban Canadians who reported using buses [31], spending more than one-hour a day on them [32].

Much of the research around IAQ in vehicles has focused on exposure to traffic-related contaminants, such as particulate matter, rather than airborne biological contaminants [4,32,33]. However, CO_2_ in vehicles can build up due to both vehicle emissions, and human breathing [4]. How fast CO_2_ builds up inside a vehicle depends on several factors, including the vehicle’s: level of occupation, air volume, and ventilation rate [4]. In urban transit vehicles, although trips are generally short, conditions (e.g., high occupation, high passenger exchange, low ventilation) can favour the propagation of airborne pathogens [4,12,26,34,35], including SARS, influenza, and RSV. Most existing standards for IAQ, such as ASHRAE standards 62.1 and 241 [13,20], apply to the control of air quality in buildings spaces. In contrast, there is a dearth of information around standards related to IAQ in ground transit vehicles, though ventilation on public transit vehicles favours natural ventilation [11].

On urban transit vehicles (i.e., buses and trams), CO_2_ monitors have been used to track temperature, relative humidity, CO_2_ concentration, and particulate matter levels across routes [21]; to assess the generation and accumulation of CO_2_ in the passenger space [11]; and to assess ventilation [26] and the factors that affect ventilation [36] in the vehicles. These studies focused on finding factors that affected the concentration of CO_2_. However, their purpose was to provide information to officials about when to implement ventilation controls, not to inform individual control strategies.

Additional studies have used CO_2_ monitors carried by individuals to assess air quality during different types of commuting [37], and during travel in different locations [25]. While these studies provided some information around personal monitoring, their intention was to inform peoples’ choices about general pollution exposure during commuting [37], or risks associated with specific travel areas [25]. The studies did not seek to empower people to take their own measurements with personal monitoring devices.

Currently, both the Canadian [38] and Ontarian [39] government recommendations for protecting against COVID-19, and other respiratory illnesses, include improving air quality and ventilation. The use of CO_2_ monitors is recommended as a strategy for improving indoor ventilation [40]; however, information around how non-experts can use them for personal risk assessment is limited [25]. Specifically, in contrast to reference (gold-standard) sensors, low cost CO_2_ monitors are not subject to evaluation and certification based on regulatory standards, so the monitor specifications, how to calibrate them, or how well they need to perform is not necessarily known [41]. Therefore, concerns exist regarding the accuracy, reliability, calibration requirements, and required correction of low-cost CO_2_ monitors [15,17,28,42]. Unreliable data can mislead users, which may contribute to the misinterpretation of contaminant levels as being acceptable or unacceptable [42].

To date, although CO_2_ monitoring has been recommended as a strategy to assess ventilation, and for risk assessment, previous studies have targeted large scale application for official use, not individual monitoring for personal risk assessment. There is some information that individual monitoring can be used to provide information related to individual exposure to particles, for example black carbon [43]. However, clear guidance is required to give non-experts confidence in using personal monitors, and to enable them to effectively use the monitors to make personal risk assessments [14,17,44].

To inform the use of CO_2_ monitors for personal risk assessment, we took measures across 250 transit trips, between October 17^th^, 2022 and July 18^th^, 2023. Using these measures, we aimed to: 1) Determine whether measurements from a low-cost CO_2_ monitor (Vitalight Mini) were consistent with an Aranet4Home CO_2_ monitor; 2) Identify factors related to the concentration of CO_2_ on urban transit buses, and determine whether they were consistent across the two CO_2_ monitors tested; and 3) Discuss the usability of a low-cost monitor, and provide some general recommendations around personal risk assessment using a monitor in combination with the evaluation of environmental factors.

## Materials and methods

### Transit system and bus fleet

For this field case study, data collection occurred on buses from the public transit authority in Greater Sudbury, the largest city in Northern Ontario, with a population of approximately 166,000. The public transit system includes ~59 standard buses, that operate over a network of 23 routes [45] and cover 1,077 stops [46]. In 2022, approximately 2.6 million trips were taken on public transit in Greater Sudbury, averaging out to about 22.7 trips per person in the service area [47]. Transit routes operate on a staggered schedule, with different routes starting and ending at different times. Operation times range between 5:30am and 12:40am on weekdays, and 6:00am and 12:30am on weekends.

**Fig 1:**
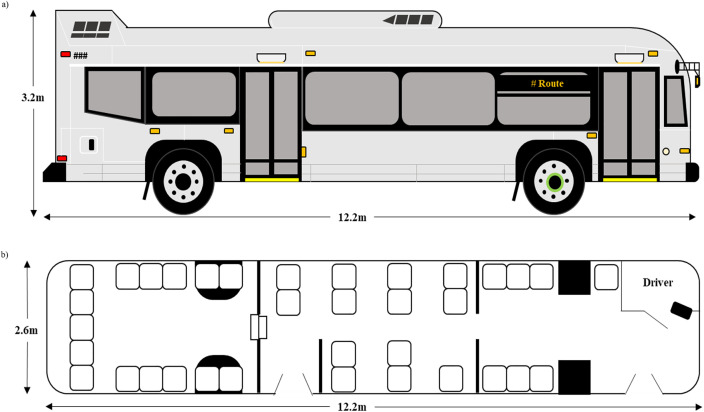
Transit bus schematic. Schematic representation of the a) exterior and b) the most common interior layout, of the GOVA transit buses (NOVA LFS diesel bus).

The bus fleet is comprised entirely of Nova LFS diesel buses, acquired between approximately 2006 and 2021. All of the standard buses are 12.2 metres long (Fig 1a), and have a maximum capacity of 80 passengers; though the interior layout of the fleet varies, one of the most common layouts is illustrated in Fig 1b. The buses have two doors on the passenger side, and 10 windows total that can be opened in addition to the driver’s window. Transit buses are equipped with an air-conditioning system (AC) that is operational in the summer at high ambient air temperature (≥25 °C), and may also be used in the spring and autumn. In the winter and the autumn, the AC is turned off, and the heating systems turned on. In general, if the AC or the heating system is turned on, the windows of the bus are closed, however, this is up to the driver’s discretion. While less likely with the heating system, it is not uncommon for windows in the passenger area to be open in combination with the AC being on.

### Ethics statement

Ethical approval and specific work permits or approvals were not required for this study as it took place in public spaces. It only involved environmental measurements, or observations of factors that did not require researchers to interact with the people in those spaces. Additionally, none of the information collected is personal information, or could be used to identify any people in those spaces.

### CO_2_ monitors

**Fig 2:**
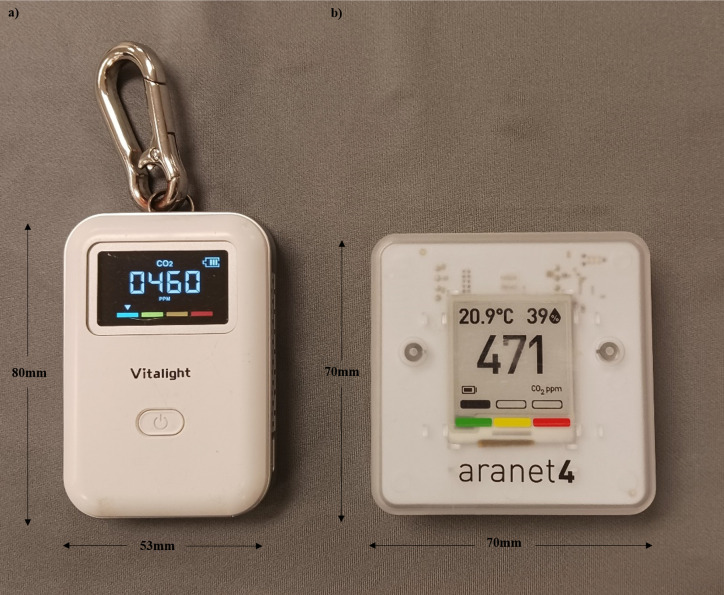
CO_2_ detectors. The **a)** Vitalight Mini CO_2_ detector, and the **b)** Aranet4 Home CO_2_ detector.

Two types of CO_2_ monitors were used, the Vitalight Mini CO_2_ Detector (Vitalight: Fig 2a) (Guangdong Bioall Medical Technology Co., LTD.: Guangdong Province, People’s Republic of China), and the Aranet4 Home (Aranet: Fig 2b) (Aranet: Riga, Latvia). The technical characteristics of the Aranet and the Vitalight are summarized in Table 1. For cost comparison, in 2022, the Vitalight was $59.99+ tax CAD on Amazon and the Aranet was $349.99+ tax CAD at Canadian Tire. Combining the cost and device characteristics (Table 1), the Vitalight was selected as it was 1/7th of the cost of the Aranet, included the non-dispersive infrared (NDIR) sensor for increased accuracy of CO_2_ measurements [15,48], operated within the desired environmental range, and had a high sampling frequency with a simple visual output similar to the Aranet. Furthermore, at the time of the study, the Vitalight was more readily available in North America, relative to other low cost monitors.

**Table 1:** Technical characteristics of the Aranet4Home detector and the Vitalight Mini CO_2_ detector.

CO_2_ Monitor	Aranet4 Home	Vitalight Mini CO_2_ Detector
**Parameters**		
**CO**_**2**_ **Concentration**	0-9999 ppm	400-5000 ppm
**Temperature**	0-50 °C (measures)	−10–50°C (operating range)
**Relative Humidity**	0-85% (measures)	0 ~ 90% (operating range)
**Accuracy CO**_**2**_	**0-5000 ppm**: ± 30 ppm; ± 3% of reading	±(50 ppm + 5% of reading)
	**>5000ppm**: ± 10% of reading	
**Sampling Frequency**	1-minute	30-120 seconds
**Sensor Type**	NDIR	NDIR
**CO**_**2**_ **Warning Display**	**Green (Good)**: 420–999 ppm	**Green**: 400–800 ppm (±50 ppm)
	**Yellow (Average)**: 1000–1400 ppm	**Yellow**: 801–1200 ppm (±50 ppm)
	**Red (Unhealthy)**: > 1400ppm	**Orange**: 1200–1500 ppm (±50 ppm)
		**Red**: > 1500ppm (±50 ppm)
**Communication Technology**	Bluetooth (4dBm or -12dBm)	N/A
**Dimensions & Weight**	70x70x24mm	53x18x80mm
	104g	78g

NDIR: Non-dispersive infrared.

For device calibration, the Vitalight performs an auto-calibration where it takes the three lowest CO_2_ readings from its previous seven days of use, and averages the value to 400 ppm [49]. Given this method of auto-calibration, for the duration of this study, the Vitalight was always turned on outside, and remained outside for approximately 30 minutes during calibration. The Aranet was used as the reference monitor in this study, as it has been found to correlate well with reference monitors in the literature [15,50]. Additionally, given that this was a field study with a long data collection period, an Aranet was considered to be more robust to constant use in a dynamic environment relative to a standard reference sensor. To compensate for this, the Aranet was calibrated by the research team in the lab against an IAQ-CALC Model 7545 (TSI Incorporated, Shoreview MN, USA) over the course of the study period. The Aranet calibrates to a baseline value of 420 ppm.

### Measurements

The Aranet and the Vitalight were both used to measure CO_2_ concentration (ppm), while the Aranet was also used to measure ambient temperature (°C), and relative humidity (%). Continuous measurement provides a more accurate assessment over time; however, while the Aranet can be used for continuous data logging, the Vitalight can only provide point-to-point measures. Therefore, a snapshot approach was used in this field study. Measurements were taken at two time points per trip, with the first measurement occurring approximately one-minute after boarding the bus (T1), and the second occurring approximately one-minute prior to disembarking (T2). Sampling took place at a height of ~0.5m (seated knee height). The date, time of day, route number, occupancy level, seating position, whether or not windows were open, number of windows open, and the number of times the bus stopped and opened the doors, were also recorded for each transit trip (Table 2).

**Table 2:** Variables measured using the ride survey, including the category and frequency of measurement per transit ride.

Variables	Recorded
**Date**	Date of ride
**Time**	Morning
	Afternoon
**Route**	Route number
**Occupancy**	Very low: < 6 passengers
	Low: 6–12 passengers
	Low-Medium: One-quarter of seats occupied
	Medium: One-half of seats occupied
	Medium-High: Three-quarters of seats occupied
	High: All seats occupied
	Very High: All seats occupied and passengers standing
**Seating Position**	Front DS
	Middle DS
	Back DS
	Back Centre
	Back PS
	Middle PS
	Front PS
**Windows Open**	No
	Yes
	Number of windows open
**Number of stops**	Number of times bus stopped and doors opened
**CO**_**2**_ **Concentration (ppm)**	Recorded at:
	Time 1: Approximately 1-minute after boarding
	Time 2: Approximately 1-minute before disembarking
**Temperature (°C)**	Recorded at:
	Time 1: Approximately 1-minute after boarding
	Time 2: Approximately 1-minute before disembarking
**Relative Humidity (%)**	Recorded at:
	Time 1: Approximately 1-minute after boarding
	Time 2: Approximately 1-minute before disembarking
**Notes**	Additional observations

DS: Driver’s side; PS: Passenger’s side; ppm: Parts per million.

Bus routes have two phases: 1) Outbound – covers when the bus leaves the terminal to when it reaches its half-way point and turns around to return; and 2) Inbound – covers when the bus turns around at the half-way point to when it returns to the terminal. Most of the 250 recorded trips were from stops at the outbound midpoint to its end, or from the start of the inbound route to its midpoint, so trips did not consist of measurements along the entire length of the route. The majority of measurements were performed during morning and afternoon commutes, and there were variable passenger loads.

### Statistical analysis

Descriptive statistics were performed on all collected variables. A two-tailed paired samples t-test was used to assess differences in CO_2_ concentration between the two monitors. Pearson correlation coefficient was used to assess the relationships between the CO_2_ concentration measured across the two monitors, and between temperature, relative humidity, and number of bus stops. With respect to correlation strength, *r*^*2*^ values < 0.5 indicated weak agreement, *r*^*2*^ values between 0.5 and 0.7 indicated moderate agreement, and *r*^*2*^ values > 0.7 indicated strong agreement [51]. Two-tailed two sample (Mann-Whitney U test) and three sample (Kruskal-Wallis H test) non-parametric tests were used to assess differences in the concentration of CO_2_ by occupancy level, seating position, and window status. For significant Kruskal-Wallis H tests, post hoc comparisons were conducted using Mann-Whitney U tests with a Bonferroni correction. All tests were performed at an alpha of.05, with the adjusted alpha for the Bonferroni correction at.0167. All statistical analyses of the data were carried out using SPSS v.29.0.1.0 (IBM Corp: Armonk, New York).

## Results

### Descriptive statistics

**Fig 3:**
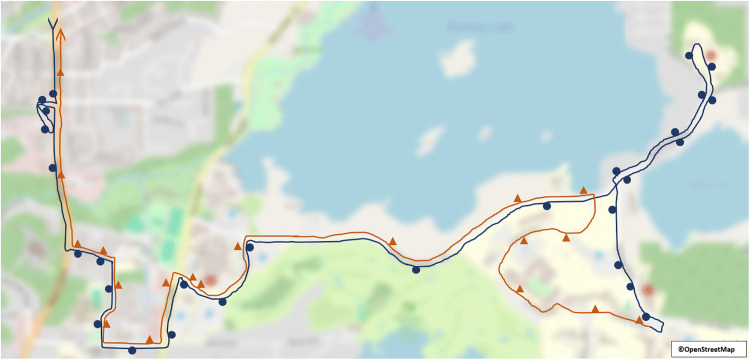
Route 3 map. Spatial map representing transit route 3, which represents 89.6% of the route data. The blue line, with the blue circle markers, represents the outbound portion of the route and its stops; the orange line, with the orange triangular markers, represents the inbound portion of the route and its stops. Spatial map was reprinted, and modified, from the original version from NASA Earth Observer [52]. The map data is licensed under the Open Data Commons Open Database License (ODbL) by OpenStreetMap and its contributors [53].

Between October 17^th^, 2022 and July 18^th^, 2023 measurements were taken across 250 transit trips, with readings most commonly taken from the front driver’s side (DS; 35.2%), the front passenger’s side (PS; 23.2%), the middle PS (22.4%), and the middle DS (12.0%) of the bus. The trips were approximately evenly split between the morning (48.8%) and the afternoon (51.2%); were predominantly carried out on route 3 (89.6%); and generally had low (34.8%), medium-low (26.0%), or medium (21.2%) levels of occupancy. Windows were only open for 15.6% of trips with an average of 2.0 (±1.3) windows observed to be open on those trips. For the 250 trips, buses made an average of 7.3 (±2.4) stops where the doors opened, with a minimum of one stop, and a maximum of 13 stops made. See Tables 3 and 4 for the complete descriptive summary, and Fig 3 for a spatial representation of the inbound and outbound portions of route 3.

**Table 3:** Descriptive summary of observations taken on the 250 transit trips between October 2022 and July 2023.

Variable		Frequency (/250)	Percentage (%)
**Time of Day**	Morning	122	48.8
	Afternoon	128	51.2
**Route#**	1	8	3.2
	3	224	89.6
	14	17	6.8
	29	1	0.4
**Occupancy**	Very low	9	3.6
	Low	87	34.8
	Medium-low	65	26.0
	Medium	53	21.2
	Medium-high	22	8.8
	High	12	4.8
	Very high	1	0.4
**Measurement Position**	Front DS	88	35.2
	Middle DS	30	12.0
	Back DS	9	3.6
	Back Centre	3	1.2
	Back PS	6	2.4
	Middle PS	56	22.4
	Front PS	58	23.2
**Windows**	Closed	211	84.4
	Open	39	15.6

DS: Driver’s side; PS: Passenger’s side.

**Table 4:** Descriptive summary of measurements taken on the 250 transit trips between October 2022 and July 2023.

Variable		N	Range	Median	IQR	Mean (±SD)
**#Open Windows**		39	1–7	2.0	1.0–2.5	2.0 (1.3)
**#Stops**		250	1–13	8.0	6.0–9.0	7.3 (2.4)
**Vitalight**	**T1 CO**_**2**_ **ppm**	250	400–1214	669.0	524.0–803.0	683.1 (185.9)
	**T2 CO**_**2**_ **ppm**	250	400–1273	623.0	515.0–736.8	644.1 (166.7)
**Aranet**	**T1 CO**_**2**_ **ppm**	250	482–1370	738.0	609.5–904.3	769.6 (189.1)
	**T2 CO**_**2**_ **ppm**	250	462–1386	696.0	606.0–829.3	735.5 (172.5)
	**T1 T C**	250	4.6–28.5	19.1	14.0–21.7	18.02 (5.00)
	**T2 T C**	250	7.5–33.9	19.8	17.1–22.1	19.66 (3.93)
	**T1 RH%**	250	12–65	31.0	23.0–41.0	32.3 (11.4)
	**T2 RH%**	250	12–61	28.0	23.0–37.0	30.2 (10.1)

T1: Time 1; T2: Time 2; ppm: Parts per million; T°C: Temperature (°C); RH%: Relative humidity (%); IQR: Interquartile Range; SD: Standard deviation.

**Fig 4:**
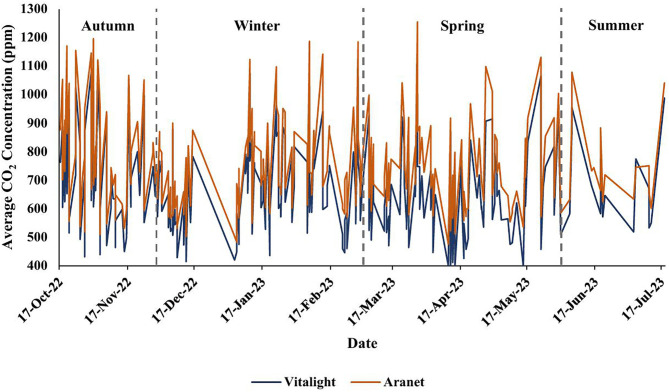
Daily average CO_2_ concentrations. Time-series plot of the daily average CO_2_ concentrations, recorded by the Vitalight and the Aranet, across the nine-month study period. Seasons are separated by dashed lines.

Fig 4 shows the daily average CO_2_ concentrations, recorded by each monitor, across the study period. At T1, CO_2_ concentrations ranged between 400–1214 ppm ((Mean ± SD) 683.1 ± 185.9) for the Vitalight, and 482–1370 ppm (769.6 ± 189.1) for the Aranet; at T2, the range was 400–1273 ppm (644.1 ± 166.7) for the Vitalight, and 462–1386 ppm (735.5 ± 172.5) for the Aranet (Table 4). Temperatures ranged from 4.6–28.5°C (18.02 ± 5.00) at T1, and 7.5–33.9°C (19.66 ± 3.93) at T2, while relative humidity was 12–65% (32.3 ± 11.4) at T1, and 12–61% (30.2 ± 10.1) at T2 (Table 4).

For the purpose of further analyses, all route data were grouped, due to the predominance of trips on route 3, compared to routes 1, 14, and 29. The occupancy and seating position variables were also re-grouped to have three categories each. New categories for occupancy were set to: 1. Low occupancy = Very low and low; 2. Medium occupancy = Medium-low and medium; and 3. High occupancy = Medium-high, high, and very high. New categories for seating position were set to: 1. Front = Front DS and front PS; 2. Middle = Middle DS and middle PS; and 3. Back = Back DS, back centre, and back PS.

### CO_2_ concentration measurements differed by type of CO_2_ monitor

**Fig 5:**
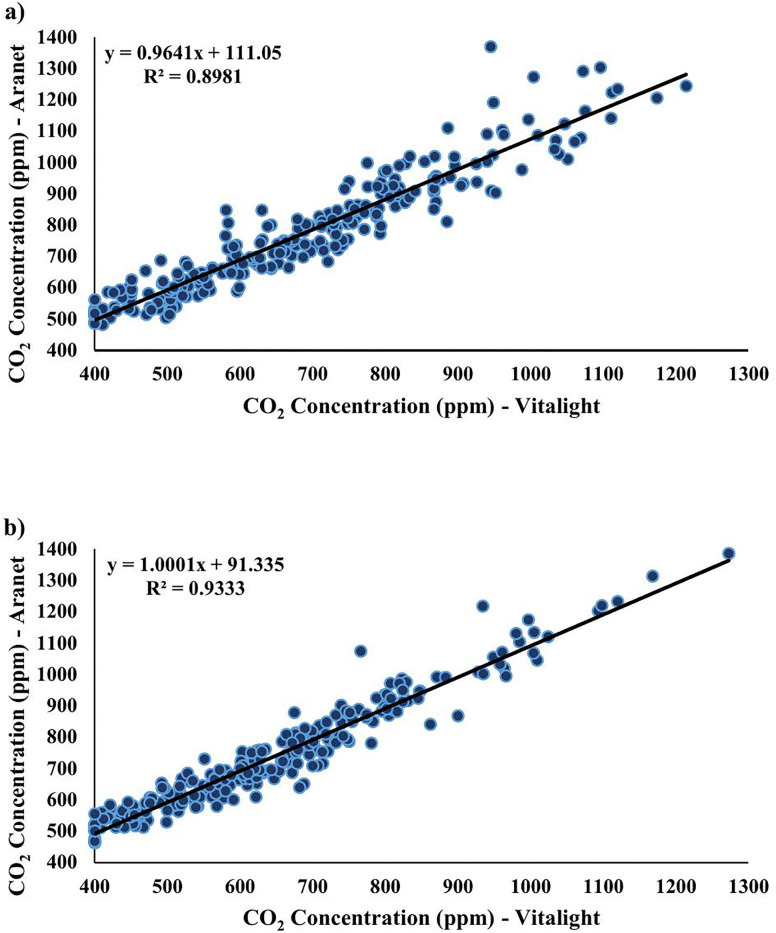
CO_2_ monitor correlation. Using Pearson’s correlation, there was a strong positive relationship between CO_2_ concentration (ppm) as measured by the Vitalight and the Aranet at a) T1 and b) T2.

Pearson correlations were performed to evaluate the relationship between the concentrations of CO_2_ measured by the Vitalight and the Aranet. There was a significant strong positive relationship between the measured concentrations of CO_2_ for the Vitalight and the Aranet, at T1 (*r*(248) =.95, p < .001) (Fig 5a), and T2 (*r*(248) =.97, p < .001) (Table 5; Fig 5b).

**Table 5:** Pearson correlations (r) between concentrations of CO_2_ measured with the Vitalight and the Aranet, and temperature, relative humidity, and number of stops with doors open, at time 1 and time 2. r^2^ values are in brackets.

T1 Variables	T1 CO_2_ Vitalight	T1 CO_2_ Aranet	T2 Variables	T2 CO_2_ Vitalight	T2 CO_2_ Aranet
**T1 CO**_**2**_ **Vitalight**	---	---	**T2 CO**_**2**_ **Vitalight**	---	---
**T1 CO**_**2**_ **Aranet**	.948** ^a^ (.899)	---	**T2 CO**_**2**_ **Aranet**	.966** ^a^ (.933)	---
**T1 T°C**	−.370** ^b^ (.137)	−.383** ^b^ (.147)	**T2 T°C**	−.033 (.00109)	−.024 (.000576)
**T1 RH%**	.519** ^b^ (.269)	.495** ^b^ (.245)	**T2 RH%**	.189** ^b^ (.0357)	.159* ^b^ (.0253)
			**T2 #Stops**	.248** ^b^ (.0615)	.267** ^b^ (.0713)

T1: Time 1; T2: Time 2; T°C: Temperature (°C); RH%: Relative humidity (%); *p < .05; **p < .01

^a^Strong correlation.

^b^Weak correlation.

**Fig 6:**
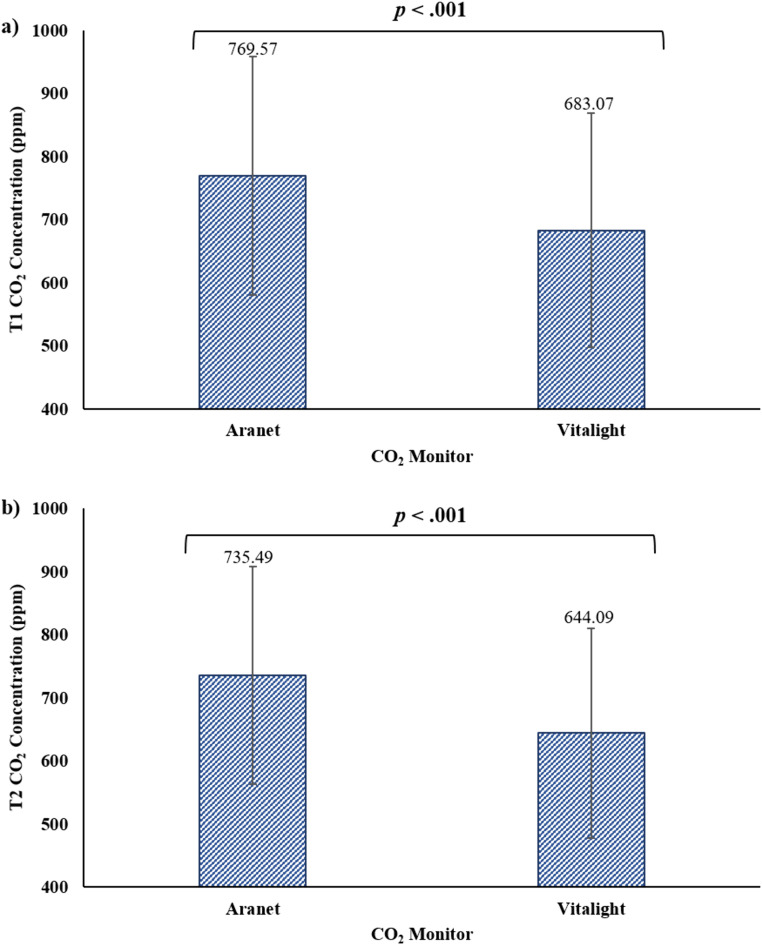
CO_2_ monitor comparison. CO_2_ concentration (ppm) as measured by the Aranet was significantly higher than the Vitalight at a) T1 and b) T2.

While measures of CO_2_ concentration were strongly correlated for the two CO_2_ monitors, two-tailed paired samples t-tests were performed to evaluate whether the measured concentration of CO_2_ differed between the Aranet and the Vitalight. At T1, the Vitalight (M = 683.07, SD = 185.862) had a significantly lower CO_2_ measurement than the Aranet (M = 769.57, SD = 189.078), *t*(249) = −22.517, p < .001 (Fig 6a). The same trend was observed at T2, where the Vitalight (M = 644.09, SD = 166.656) had a significantly lower CO_2_ measurement than the Aranet (M = 735.49, SD = 172.525), *t*(249) = −32.436, p < .001 (Fig 6b).

### CO_2_ concentration is negatively correlated to temperature at T1

Pearson correlations were performed to evaluate the relationship between the concentration of CO_2_ and temperature. There was a significant weak negative relationship between the concentration of CO_2_ and temperature at T1, as measured by the Vitalight (*r*(248) = −.37, p < .001), and the Aranet (*r*(248) = −.38, p < .001) (Table 5). The relationship between the concentration of CO_2_ and temperature at T2 was not significant for the Vitalight (*r*(248) = −.03, p = .606), or the Aranet (*r*(248) = −.02, p = .704) (Table 5).

### CO_2_ concentration is positively correlated to relative humidity at T1 and T2

Pearson correlations were performed to evaluate the relationship between the concentration of CO_2_ and relative humidity. There was a significant weak positive relationship between the concentration of CO_2_ and relative humidity at T1 for the Vitalight (*r*(248) =.52, p < .001) and the Aranet (*r*(248) =.50, p < .001), and at T2 for the Vitalight (*r*(248) =.19, p = .003), and the Aranet (*r*(248) =.16, p = .012) (Table 5).

### CO_2_ concentration is positively correlated to the number of times the bus stopped

Pearson correlations were performed to evaluate the relationship between the concentration of CO_2_ and the number of stops with doors open that the bus made. The relationship between the concentration of CO_2_ and the numbers of stops was only assessed at T2, as only at this time point had the maximum number of stops been made. There was a significant weak positive relationship between the concentration of CO_2_ and the number of stops the bus made for both the Vitalight (*r*(248) =.25, p < .001), and the Aranet (*r*(248) =.27, p < .001) (Table 5).

### CO_2_ concentration differed by occupancy level at T2

**Fig 7:**
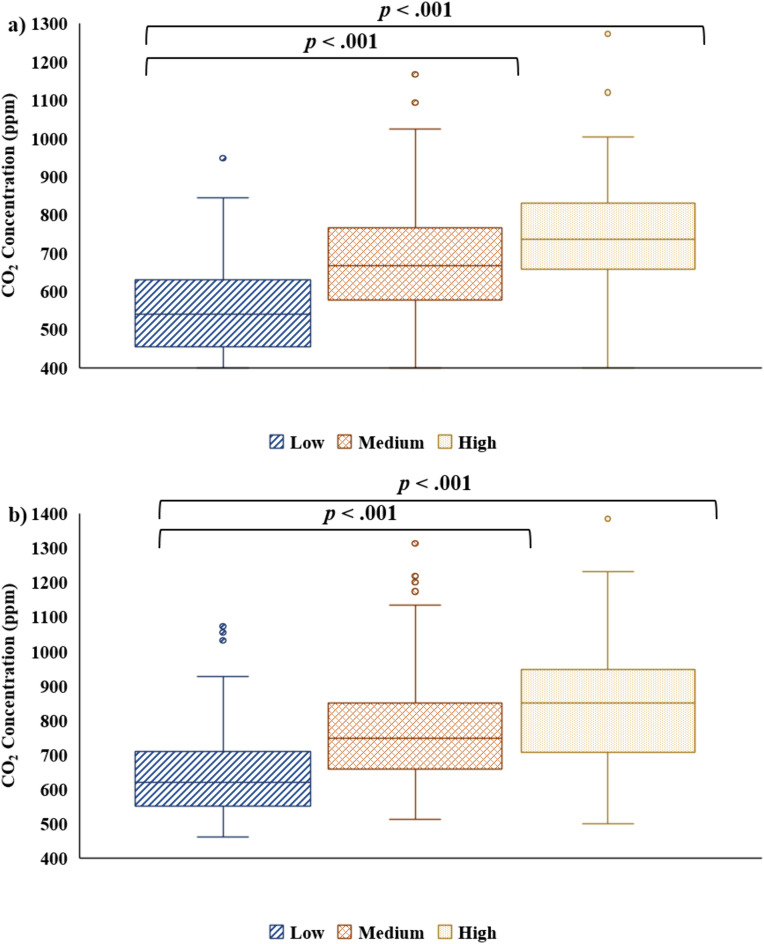
CO_2_ concentration by occupancy. CO_2_ concentration (ppm) was significantly affected by occupancy level at T2, according to the Kruskal-Wallis H test. Mann-Whitney U post hoc tests showed CO_2_ was lower at low compared to medium and high occupancy, as measured by a) the Vitalight and b) the Aranet.

Kruskal-Wallis tests were performed to evaluate whether the concentration of CO_2_ differed by occupancy level. The results indicated that there was a significant difference in the concentration of CO_2_ measured by the Vitalight at T2 at low, medium, and high occupancy, *H*(2) = 50.345, p < .001. Mann-Whitney U post hoc comparisons with a Bonferroni adjustment indicated that the concentration of CO_2_ measured by the Vitalight at T2 was significantly lower at low occupancy (*Mdn* = 540.5, *IQR* = 453.8–629.3) compared to medium occupancy (*Mdn* = 668.0, *IQR* = 577.8–765.8) (U = 2860.00, z = −6.22, p < .001), and at low occupancy (*Mdn* = 540.5, *IQR* = 453.8–629.3) compared to high occupancy (*Mdn* = 736.0, *IQR* = 657.0–831.0) (U = 676.00, z = −5.224, p < .001) (Fig 7a). The other comparison was not statistically significant after the Bonferroni adjustment (p > .0167). Similarly, there was a significant difference in the concentration of CO_2_ measured by the Aranet at T2 at low, medium, and high occupancy, *H*(2) = 46.570, p < .001. Mann-Whitney U post hoc comparisons with a Bonferroni adjustment indicate that the concentration of CO_2_ measured by the Aranet at T2 was significantly lower at low occupancy (*Mdn* = 620.0, *IQR* = 551.0–710.0) compared to medium occupancy (*Mdn* = 747.5, *IQR* = 659.3–850.8) (U = 2985.00, z = −5.95, p < .001), and at low occupancy (*Mdn* = 620.0, *IQR* = 551.0–710.0) compared to high occupancy (*Mdn* = 849.0, *IQR* = 708.0–947.0) (U = 712.50, z = −5.033, p < .001) (Fig 7b). The other comparison was not statistically significant after the Bonferroni adjustment (p > .0167). At T1, there was no significant difference in the concentration of CO_2_ measured by the Vitalight at low (*Mdn* = 678.0, *IQR* = 544.8–796.3), medium (*Mdn* = 665.0, *IQR* = 524.5–815.0), or high (*Mdn* = 638.0, *IQR* = 503.0–841.0) occupancy, *H*(2) = 0.163, p = .922, or by the Aranet at low (*Mdn* = 736.0, *IQR* = 607.0–888.8), medium (*Mdn* = 734.5, *IQR* = 604.5–916.3), or high (*Mdn* = 796.0, *IQR* = 613.0–910.0) occupancy, *H*(2) = 0.108, p = .947.

### CO_2_ concentration differed by seating position at T1

**Fig 8:**
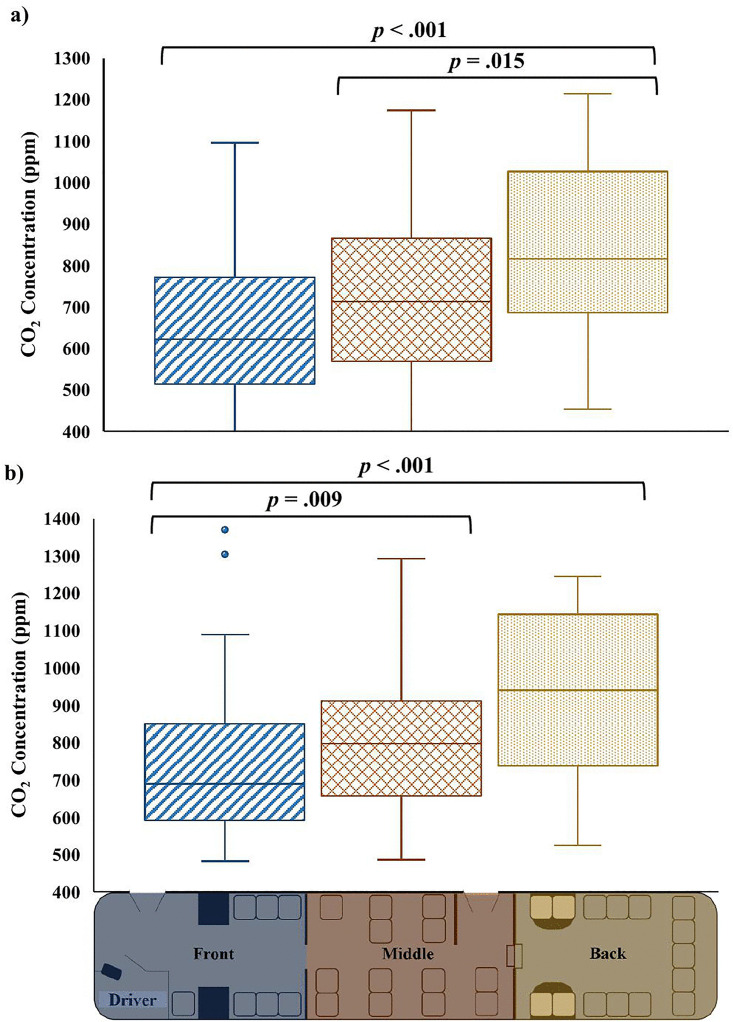
CO_2_ concentration by seating position. CO_2_ concentration (ppm) was significantly affected by seating position at T1, according to the Kruskal-Wallis H test. Mann-Whitney U post hoc tests showed CO_2_ was higher at the back of the bus compared to the front and the middle, as measured by a) the Vitalight, and lower at the front of the bus compared to the middle and the back, as measure by b) the Aranet.

Kruskal-Wallis tests were performed to evaluate whether the concentration of CO_2_ differed by seating position. There was a significant difference in the concentration of CO_2_ measured by the Vitalight at T1 between the front, middle, and back seating positions, *H*(2) = 17.130, p < .001. Mann-Whitney U post hoc comparisons with a Bonferroni adjustment indicate that the concentration of CO_2_ was significantly lower in the front of the bus (*Mdn* = 622.0, *IQR* = 513.8–771.0) compared to the back of the bus (*Mdn* = 816.0, *IQR* = 686.0–1026.3) (U = 591.00, z = −3.804, p < .001), and in the middle of the bus (*Mdn* = 713.0, *IQR* = 569.3–866.3) compared to the back of the bus (*Mdn* = 816.0, *IQR* = 686.0–1026.3) (U = 491.00, z = −2.428, p = .015) (Fig 8a). The other comparison was not statistically significant after the Bonferroni adjustment (p > .0167). Likewise, there was a significant difference in the concentration of CO_2_ measured by the Aranet at T1 between the front, middle, and back seating positions, *H*(2) = 17.466, p < .001. Mann-Whitney U post hoc comparisons with a Bonferroni adjustment indicate that the concentration of CO_2_ was significantly lower in the front of the bus (*Mdn* = 689.5, *IQR* = 591.8–849.8) compared to the middle of the bus (*Mdn* = 796.5, *IQR* = 657.3–911.5) (U = 4988.50, z = −2.612, p = .009), and in the front of the bus (*Mdn* = 689.5, *IQR* = 591.8–849.8) compared to the back of the bus (*Mdn* = 940.0, *IQR* = 737.5–1143.8) (U = 617.50, z = −3.664, p < .001) (Fig 8b). The other comparison was not statistically significant after the Bonferroni adjustment (p > .0167). At T2, there was not a significant difference in the concentration of CO_2_ measured by the Vitalight between the front (*Mdn* = 612.0, *IQR* = 511.3–718.3), middle (*Mdn* = 652.5, *IQR* = 511.0–740.5), or back (*Mdn* = 699.0, *IQR* = 568.5–964.5) seating positions, *H*(2) = 4.330, p = .115, or by the Aranet between the front (*Mdn* = 692.5, *IQR* = 597.3–810.8), middle (*Mdn* = 723.0, *IQR* = 614.8–818.0), or back (*Mdn* = 772.5, *IQR* = 650.5–1006.3) seating positions, H(2) = 3.563, p = .168.

### CO_2_ concentration differed by window status at T1 and T2

**Fig 9:**
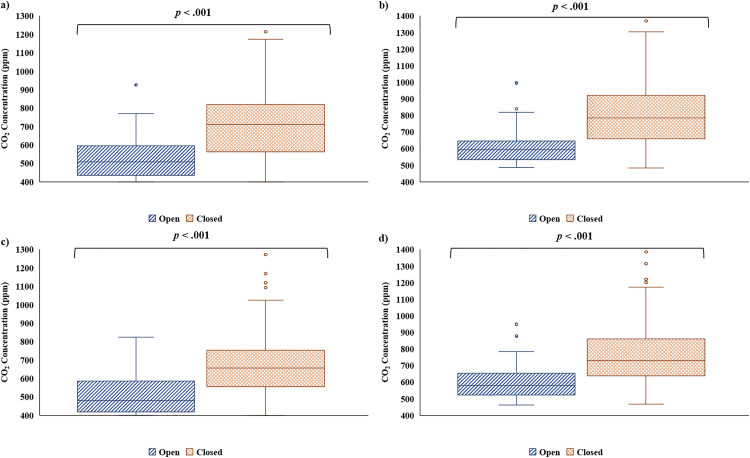
CO_2_ concentration by window status. CO_2_ concentration (ppm) was significantly higher when the bus windows were closed compared to open, as measured by a) the Vitalight, and b) the Aranet, at T1, and c) the Vitalight, and d) the Aranet, at T2, according to Mann-Whitney U tests.

Two-tailed Mann-Whitney U tests were performed to evaluate whether the concentration of CO_2_ differed when the windows on the bus were open or closed. At T1, the concentration of CO_2_ measured by the Vitalight was significantly lower when windows were open (*Mdn* = 509.0, *IQR* = 435.0–596.0) compared to closed (*Mdn* = 712.0, *IQR* = 562.0–819.0), U = 1710.50, z = −5.80, p < .001 (Fig 9a). Likewise, the concentration of CO_2_ measured by the Aranet was significantly lower when windows were open (*Mdn* = 594.0, *IQR* = 533.0–647.0) compared to closed (*Mdn* = 786.0, *IQR* = 660.0–920.0), U = 1710.50, z = −5.80, p < .001 (Fig 9b). The same trend was observed at T2, where the concentration of CO_2_ as measured by the Vitalight was significantly lower when windows were open (*Mdn* = 479.0, *IQR* = 419.0–586.0) compared to closed (*Mdn* = 657.0, *IQR* = 555.0–754.0), U = 1648.50, z = −5.94, p < .001 (Fig 9c). Similarly, the concentration of CO_2_ measured by the Aranet was significantly lower when windows were open (*Mdn* = 579.0, *IQR* = 523.0–655.0) compared to closed (*Mdn* = 730.0, *IQR* = 639.0–862.0), U = 1557.00, z = −6.17, p < .001 (Fig 9d).

## Discussion

### Rebreathed air

Ongoing concerns related to airborne infectious illnesses have increased the focus on using CO_2_ monitors as a proxy for infection risk [10,11], as both CO_2_ and airborne contaminants can build up if there’s insufficient ventilation [4,24]. Public transit vehicles, can represent a health risk to drivers, and passengers, as contacts between people facilitate exposures to airborne biological contaminants [29].

In this work, CO_2_ measurements were collected across 250 transit trips, with an average trip duration of 15−20 minutes. The average concentration of CO_2_ measured with the Aranet was 769.6 ± 189.1 ppm (Vitalight: 683.1 ± 185.9) at T1, and 735.5 ± 172.5 (Vitalight: 644.1 ± 166.7) at T2. Carbon dioxide is predominantly produced by the occupants in indoor spaces, so its concentration is representative of the fraction of rebreathed air [14–16]. Therefore, based on the Aranet values, on average the proportion of rebreathed air during these commutes was ~ 0.7–1.0% (Vitalight: ~ 0.5–0.7%) [22]. However, with measures of CO_2_ ranging between 482−1370 ppm for the Aranet (Vitalight: 400−1214) at T1, and 462−1386 (Vitalight: 400−1273) at T2, the proportion of rebreathed air could range from ~0%−2.6% (Vitalight: ~ 0–2.3%), such that up to every one in 39 breaths were considered rebreathed [22]. Combining the typical adult respiratory rate of 12−20 breaths per minute [54], with 1/39 rebreathed breaths at the documented CO_2_ concentrations indicated in a 15−20 minute trip, an occupant could rebreathe between 6.2 to 10.3 contaminated breaths. As concentrations of CO_2_ above 800 ppm increase SARS-CoV-2 aerostability [10], this study indicates that, at times, the measures of CO_2_ were high enough to suggest increased concern for transmissibility of this airborne hazard.

### Are measures of CO_2_ concentration consistent between the Vitalight and the Aranet?

Low-cost CO_2_ monitors are recommended as a potential strategy for improving indoor ventilation [40]; however, information around how non-experts can use them for personal risk assessment is limited [25]. Therefore, the first objective of this paper was to compare the performance of a Vitalight CO_2_ monitor against an Aranet, as the Vitalight is more budget friendly to the general public, however, it lacks the published performance validation of the Aranet [15,50].

Ranges of CO_2_ concentration as measured by the Vitalight (400–1273 ppm) and the Aranet (462–1386 ppm) were consistent with values measured on urban transit vehicles in Brunswick and Hanover, Germany (Bus: 400–1161 ppm; Tram: 400–1197 ppm; Train: 400–1105 ppm) [21]; Cleveland, United States (Articulated bus peak CO_2_ at peak and non-peak occupancy: 760 and 790 ppm; Standard bus peak CO_2_ at peak and non-peak occupancy: 1600 and 920 ppm; Shuttle van peak CO_2_ at peak and non-peak occupancy: 1870 and 1360 ppm) [26], Zaragoza, Spain (Tram: 572 ± 75–835 ± 232 ppm) [11]; and Barcelona, Spain (Three types of buses: 555–2112 ppm) [36]. However, with respect to this study, compared to the Aranet, the Vitalight tended to underrepresent the CO_2_ concentration, though the monitor measures were highly correlated. These observations are consistent with the literature, where both Ishigaki and colleagues [15] and Demanega and colleagues [48] found that low-cost CO_2_ monitors that used NDIR sensors, correlated well with reference monitors, and could be used to indicate trends in CO_2_, if they were properly calibrated. Therefore, although the absolute values measured by the Vitalight and the Aranet are not exactly the same, the Vitalight is capable of dynamically tracking changes in CO_2_ concentration, in a trend consistent with the Aranet.

### Factors related to the concentration of CO_2_ across monitors

In addition to determining whether CO_2_ measures were consistent between the Vitalight and the Aranet, we wanted to identify other factors that could affect CO_2_ concentration. Therefore, the second objective of this paper was to identify factors related to the concentration of CO_2_ on transit buses, and determine if those factors were consistent across monitors.

Factors that were previously found to affect the measurements of CO_2_ include: relative humidity and temperature [21,41,55,56]; open windows or doors [6,34–36,57]; level of occupancy [6,11,21,35,37]; and seating position or room location [6,36,58,59]. In this study, these factors were assessed to determine if responses to them were consistent across monitors. For all of the factors assessed, the Vitalight paralleled the Aranet in response direction.

NDIR sensors can respond to environmental factors such as temperature and relative humidity, as they can change the properties of the gas molecules that the sensors assess [55,60]. In the current study, both the Vitalight and the Aranet showed a weak negative correlation with temperature at T1, indicating that at this time point as temperature increased, the measured concentration of CO_2_ decreased. At T2, no significant correlation with temperature was found with either monitor. For relative humidity, there was a weak positive correlation for both monitors, indicating that as relative humidity increased, the measured concentration of CO_2_ increased. The correlation was significant at both time points, however, the *r* values for both monitors decreased from T1 to T2. Consistent with the current study, a simulation study done to identify environmental factors that affect CO_2_ sensor accuracy and precision found that when temperature increases, the measure of CO_2_ decreases, and when relative humidity increases, the measure of CO_2_ increases [61]. These observations are also consistent with a study assessing field calibration methods, that found CO_2_ measures were negatively correlated with temperature (r = −.61) and positively correlated with relative humidity (r = .62) [56]. In the current study, higher correlations of temperature and relative humidity were observed at T1 compared to T2, which may indicate a holdover effect on the monitors in changing from outdoor ambient conditions to indoor conditions. In-line with this hypothesis, Ródenas García and colleagues [41] observed that a warm-up time may be required to ensure a sensor’s optical system is thermally stable in order to improve their accuracy. In general, field calibration, or calibration of the monitors to the conditions they are going to be used at, can help to improve their performance accuracy [62,63].

Ventilation on public transit vehicles favours natural ventilation [11], which includes opening the windows and doors. In the current study, for both the Vitalight and the Aranet, the concentration of CO_2_ was significantly lower when the bus windows were open, compared to closed, and this was observed at both T1 and T2. Consistent with these observations, opening the windows in buildings [6], and driving with the windows open in commercial trucks [64], school buses [65], tour buses [58], and public transit buses [34,36,57], has been shown to reduce the measured concentration of CO_2_. For example, an assessment of air change rates found that in buses with open windows, the number of air changes per hour was more than 2.5 times higher than in buses with closed windows [57]. Similarly, both Baselga and colleagues [11] and Zhu and colleagues [35] found that wind speed affects the rate of natural ventilation, with higher wind speeds leading to higher ventilation. However, as wind speed was not assessed in the current study, it cannot be determined whether this trend holds true with these data. In contrast to opening the windows, for both the Vitalight and the Aranet, there was a weak positive correlation with the number of times the doors on the bus opened, indicating that higher levels of CO_2_ tended to occur when the doors opened more. These observations are somewhat opposed to studies in the literature that show opening vehicle doors helped to reduce levels of CO_2_ [24,35,36]. This discrepancy may reflect that, in the current study, when the doors on the bus opened more, the bus occupancy did not necessarily change sufficiently to decrease CO_2_ concentrations. Additionally, when the bus stopped, both sets of doors did not always open, which could also have limited their role in air exchange during stops. It is possible that outdoor CO_2_ concentrations could have been elevated while the bus doors opened, but given the rural study location, this is unlikely to have affected the concentration gradient in short bursts.

In indoor spaces, CO_2_ is largely produced by the occupants of the space, so CO_2_ concentration can provide information around how crowded a space is [2,14]. On crowded buses, CO_2_ levels have been observed to be three times higher than ambient outdoor concentrations (i.e., 1200 ppm vs. 400 ppm) [37]. In the current study, for both the Vitalight and the Aranet, at T2 the concentration of CO_2_ was lower at low occupancy compared to both medium and high occupancies. These data are consistent with observations in other types of passenger vehicles, including cars [66], buses, such as shuttle buses, articulated buses, and standard buses [21,26,35,37], trams [11,21], and trains [21], as well as in buildings [6]. In all these cases, high levels of occupancy have been related to higher concentrations of CO_2_, as well as a reduced amount of time needed to reach high CO_2_ concentrations [66]. The concentration of CO_2_ did not differ across levels of occupancy for either monitor at T1, however, this is likely a limitation in the methodology. An estimate of average occupancy was only taken once, as immediately following boarding, both in the morning and the afternoon, there were large fluctuations in occupancy as there are multiple close placed stops at these points in the route, so accurate estimates were difficult to make. In contrast, closer to T2, stops tend to be spaced further apart so occupancy is more static. Therefore, the occupancy estimates were ultimately more reflective of occupancy at T2 rather than T1.

The distribution of gases, such as CO_2_, within the passenger space of transit vehicles is heterogeneous, and dependent on patterns of airflow within the space [59,67]. Observations with the Vitalight and the Aranet indicate that there were differences in the dispersion of CO_2_ along the length of the bus noted at T1. For the Vitalight, concentrations of CO_2_ were significantly higher at the back of the bus, relative to the middle section and the front section; for the Aranet, concentrations of CO_2_ were significantly lower in the front of the bus, relative to the middle section and the back sections. Though there was a slight discrepancy between the Vitalight and the Aranet, in general, measured concentrations were lowest at the front of the bus, and highest at the back of the bus. Similar dispersions have been noted in tour buses [58], and transit buses, as drivers tend to keep their windows open, contributing to lower CO_2_ concentrations in the front of the bus, while the absence of doors in the far back of the buses contributes to higher concentrations [36]. The concentration of CO_2_ did not differ across seating position for either monitor at T2. The different influence of seating position on concentrations of CO_2_ between T1 and T2 is not specifically known, but different levels of air flow at the two time points could contribute. For example, at T1 there are multiple closely placed stops, relative to T2, where stops tend to be spaced further apart leading to more uninterrupted driving time. Longer spans of uninterrupted driving time, could facilitate more airflow, through open windows, as well as through “leaks” in door gaps or poorly sealed windows. This could contribute to a more homogenous CO_2_ concentration throughout the length of the bus, in contrast to stop and go driving.

### Usability of the Vitalight monitor

Building on the previous two objectives, the final objective of this study was to assess the usability of the Vitalight. Additionally, we provide some general recommendations around personal risk assessment using the Vitalight in combination with the evaluation of environmental factors.

Overall, the data in this study indicate that measurements taken with the Vitalight were highly correlated to those taken by the Aranet at both T1 and T2. Furthermore, the Vitalight paralleled the Aranet in measurement responses to: temperature, relative humidity, number of stops made, occupancy level, seating position, and whether windows were open or closed. The largest discrepancy between the two monitors was the absolute measured values of CO_2_, such that the Vitalight tended to underrepresent the concentration relative to the Aranet. Taken together, these data indicate that the Vitalight is capable of dynamically tracking changes in CO_2_ concentration, so increases and decreases in concentration can be evaluated in real-time.

Consistent with the observations in this study, Ishigaki and colleagues [15] assessed the accuracies of 12 low cost, and seven high cost, CO_2_ sensors, and they found that the NDIR sensors that correlated well with a reference sensor, could be calibrated and used to identify trends in CO_2_ concentration. Although multiple methods of calibration exist (e.g., [9,41,55,56,68]), given that the purpose of the current study is to outline usability of a low-cost CO_2_ monitor for non-experts, we have suggested recommendations that are simple, and useable by a wide audience, without a specific correction factor for the Vitalight.

**Fig 10:**
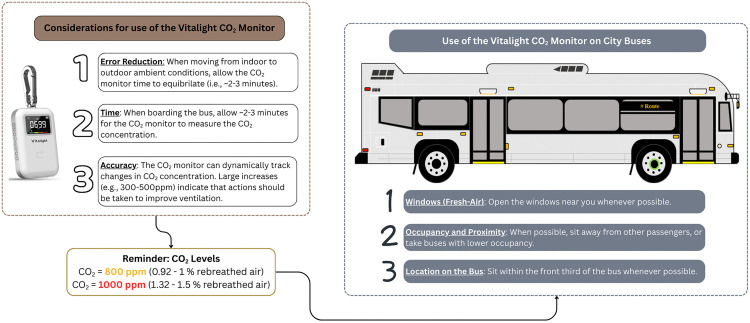
Recommendation summary. Summary of the three recommendations for personal risk assessment using the Vitalight to assess ventilation.

Taken together, there are three main recommendations (Fig 10) around personal risk assessment that can be extracted from these results:

1. To reduce error on readings when moving from outdoor ambient conditions to indoor conditions, the Vitalight should be allowed a brief “warm-up” time to allow it to equilibrate to the new temperature and humidity conditions. The holdover effect of external temperature and humidity was observable at T1, or approximately 1-minute post boarding, so a warm-up time of several minutes is recommended;
2. The Vitalight can dynamically track changes in CO_2_ concentration, and large increases (e.g., 300–500 ppm) indicate that actions should be taken to improve ventilation. For example, if the initial measurement is 500 ppm, or approximately that of outdoor air, an increase between 300–500 ppm would put the measurement in the range of 800–1000 ppm which is high, indicating that ventilation should be improved;
3. When CO_2_ readings are high (800 ppm or above [3,14,26]), or to keep them from getting high, transit riders can:
  - Open the windows on the bus;
  - Sit away from other passengers, or, if possible, take buses with lower levels of occupancy or at less busy times of day;
  - Sit closer to the front of the bus, preferentially within the front third.

Overall, these recommendations can help people to use CO_2_ readings from the Vitalight to estimate whether ventilation on transit buses is sufficient, as well as providing people with actions they can take to keep readings low.

### Limitations

This field study is not intended to provide exhaustive information on the average concentration of CO_2_ on transit buses in Sudbury. Instead, it was meant to assess the real-world usability of a non-validated low-cost CO_2_ monitor for personal monitoring, and to determine additional environmental factors that could be controlled to reduce readings, so not all factors in the transit space were controlled. A small number of bus routes out of the entire network were evaluated, and measurements were taken during prime commuting times in the morning and the afternoon. Additionally, aside from whether the bus windows were open or closed, no data was collected about the ventilation settings on the buses, such as whether the air was being recirculated.

Air quality measures are not precise, and characteristics like temperature, humidity, airflow, the composition of contaminants in the air, etc., are fluid, and change over time [14,69]. Therefore, any measures for air quality are relative to the time and conditions when the measure was taken [69]. Additionally, information from CO_2_ monitors is dependent on the accuracy of the monitor used, the location of the monitor, and specific monitor calibration requirements [14,17]. For this data, a limitation was the snapshot approach, which was used due to the Vitalight monitor’s inability to continuously data log. Consequently, the data here reflect the specific CO_2_ monitors used, the conditions of the commuting trips taken, and the time points when the data was collected.

Ideally, CO_2_ monitors should be placed ~ 0.5m away from windows, doors, ventilation grilles, and people [3,14,28], however, on a bus this is not reasonably feasible. Placing CO_2_ monitors in individual’s breathing zones can increase readings relative to those that are further away from occupants [24], which may have impacted CO_2_ measurements taken with both monitors in this study.

Another limitation is that the Vitalight performs an auto-calibration where it takes the three lowest CO_2_ readings from its previous seven days of use, and averages the value to 400 ppm [49]. Given this method, the Vitalight monitor was always turned on outside, to ensure calibration to the outdoor ambient concentration of CO_2_. However, using 400 ppm as the baseline concentration can be inaccurate, because, even outside, the concentration of CO_2_ varies and can be higher than 400 ppm. For example, average CO_2_ concentrations measured outside of four schools in Ottawa, Ontario, in the fall of 2013, ranged between 389 ppm (95% CI 374–403 ppm) and 842 ppm (95% CI 386–1298 ppm) [70]. Calibration to the incorrect baseline, will affect the reliability of all measured data, which could limit the usability of the Vitalight in locales where the ambient CO_2_ concentration is significantly different from 400 ppm.

While the above factors may have limited the accuracy of the collected data, the paralleled responses of two different CO_2_ monitors across field conditions demonstrate that low-cost CO_2_ monitors can dynamically assess changes in CO_2_, indicating that they can be used as a component of a personal monitoring strategy regarding the sufficiency of ventilation in transit vehicles.

## Conclusions

Measurements of CO_2_ and surveying of environmental factors were carried out with two CO_2_ monitors during commutes on 250 public transit trips, with the aim of assessing the usability of a low-cost CO_2_ monitor, and providing general recommendations around personal risk assessment using a low-cost monitor in combination with the evaluation of environmental factors. The results show that CO_2_ measurements taken with the Vitalight were highly correlated with those taken by the Aranet, and the Vitalight paralleled the Aranet with respect to its responses to: temperature; relative humidity; occupancy level; seating position; how often the bus doors opened; and whether the windows were open or closed. However, measurements of CO_2_ taken by the Vitalight, on average, underrepresented measures taken by the Aranet. Based on these observations, a list of recommendations and actions related to the use of the Vitalight for personal risk assessment was developed.

Future research should look at evaluating additional Vitalight monitors to assess the precision of the CO_2_ sensors across individual devices. Additionally, as the purpose of this study was to provide recommendations to non-experts to facilitate their use of low-cost monitors in personal risk assessment, future studies should assess the usability of these recommendations by non-experts, and whether there are associated behaviour changes, such as increased confidence and self-efficacy, with their application in personal monitoring.

## Supporting information

S1 Table Data. Full study data set.
(XLSX)

S2 Table Extreme Trip Data. Subset of data for the trips with peak CO_2_ and occupancy levels.
(DOCX)
