# Supplementary material for: Determining the reliability of the Vitalight CO2 Monitor via the assessment of the carbon dioxide profile on city transit buses
Source: PLoS One. 2026 Feb 11;21(2):e0342566. doi: 10.1371/journal.pone.0342566 (PMC12893571; doi:10.1371/journal.pone.0342566)
Supplement: S2 Table — Subset of data for the trips with peak CO2 and occupancy levels. (DOCX) [file pone.0342566.s002.docx]

**S2. Extreme Trip Data**

**Table 1.** Data coding

| **Date** | **Time** | **Occupancy** | **Windows** | **Position on Bus** | **All Variables** | **Short Forms** |
| --- | --- | --- | --- | --- | --- | --- |
| MM.DD.YY | 0 – Morning  1 – Afternoon  2 – Evening | 0 – Very low  1 – Low  2 – Low-medium  3 – Medium  4 – Medium-high  5 – High  6 – Very high | 0 – Open  1 – Closed | 0 – Front left  1 – Middle left  2 – Back left  3 – Back centre  4 – Back right  5 – Middle right  6 – Front right | 999 – No response | V_[CO2]_T1 – Vitalight CO_2_ measurement at time 1  V_[CO2]_T2 – Vitalight CO_2_ measurement at time 2  A_[CO2]_T1 – Aranet CO_2_ measurement at time 1  A_[CO2]_T2 – Aranet CO_2_ measurement at time 2  Temp1 – Temperature at time 1  Temp2 – Temperature at time 2  RH1 – Relative humidity at time 1  RH2 – Relative humidity at time 2  Occup. – Occupancy  Wind. – Windows  #Wind. – Number of windows open  Posit. – Position on the bus |

**Table 2.** Extreme trip data

| **Ride#** | **Date** | **Time** | **V_[CO2] _T1** | **V_[CO2] _T2** | **A_[CO2] _T1** | **A_[CO2] _T2** | **Temp1** | **Temp2** | **RH1** | **RH2** | **Occup.** | **Wind.** | **#Wind.** | **#Stops** | **Posit.** |
| --- | --- | --- | --- | --- | --- | --- | --- | --- | --- | --- | --- | --- | --- | --- | --- |
| R2 | 10.18.22 | 0 | 949 | 831 | 1191 | 916 | 16.3 | 20.2 | 49 | 45 | 5 | 1 | 999 | 13 | 1 |
| R6 | 10.20.22 | 0 | 945 | 807 | 1370 | 971 | 13.1 | 20.3 | 51 | 43 | 4 | 1 | 999 | 10 | 6 |
| R13 | 10.24.22 | 1 | 776 | 1273 | 923 | 1386 | 25.3 | 20.2 | 31 | 40 | 4 | 1 | 999 | 10 | 0 |
| R21 | 10.31.22 | 0 | 988 | 1168 | 977 | 1314 | 16.7 | 19.9 | 51 | 52 | 3 | 1 | 999 | 9 | 0 |
| R23 | 11.01.22 | 0 | 1004 | 1024 | 1273 | 1120 | 17.4 | 21.5 | 45 | 42 | 3 | 1 | 999 | 11 | 1 |
| R27 | 11.03.22 | 0 | 1112 | 964 | 1224 | 1019 | 20.0 | 21.9 | 53 | 49 | 2 | 1 | 999 | 12 | 2 |
| R44 | 11.17.22 | 1 | 907 | 1093 | 933 | 1202 | 15.7 | 14.9 | 24 | 27 | 3 | 1 | 999 | 8 | 2 |
| R96 | 01.11.23 | 0 | 1214 | 935 | 1245 | 1002 | 12.1 | 16.6 | 29 | 24 | 4 | 1 | 999 | 7 | 3 |
| R99 | 01.12.23 | 1 | 503 | 519 | 515 | 584 | 17.0 | 15.4 | 23 | 30 | 4 | 1 | 999 | 9 | 0 |
| R111 | 01.23.23 | 0 | 940 | 985 | 1091 | 1104 | 19.8 | 17.7 | 34 | 33 | 4 | 1 | 999 | 10 | 5 |
| R112 | 01.23.23 | 1 | 727 | 980 | 786 | 1131 | 22.7 | 21.3 | 20 | 24 | 4 | 1 | 999 | 7 | 4 |
| R121 | 01.30.23 | 1 | 471 | 633 | 515 | 690 | 20.8 | 18.2 | 13 | 17 | 5 | 1 | 999 | 8 | 5 |
| R126 | 02.07.23 | 0 | 1096 | 961 | 1304 | 1071 | 16.6 | 20.0 | 47 | 38 | 2 | 1 | 999 | 8 | 6 |
| R147 | 03.01.23 | 0 | 1120 | 1005 | 1235 | 1134 | 11.2 | 21.8 | 47 | 26 | 2 | 1 | 999 | 8 | 3 |
| R167 | 03.21.23 | 0 | 997 | 847 | 1137 | 946 | 5.5 | 13.0 | 47 | 35 | 3 | 1 | 999 | 7 | 2 |
| R176 | 03.28.23 | 0 | 1072 | 934 | 1292 | 1218 | 11.2 | 20.1 | 35 | 26 | 3 | 1 | 999 | 7 | 5 |
| R188 | 04.12.23 | 0 | 1111 | 627 | 1142 | 694 | 23.6 | 33.9 | 30 | 17 | 2 | 1 | 999 | 10 | 1 |
| R189 | 04.12.23 | 1 | 400 | 400 | 525 | 520 | 28.5 | 26.4 | 27 | 30 | 4 | 0 | 4 | 5 | 0 |
| R191 | 04.13.23 | 1 | 400 | 422 | 562 | 571 | 28.2 | 29.1 | 30 | 28 | 4 | 0 | 3 | 10 | 0 |
| R209 | 04.28.23 | 0 | 1047 | 766 | 1123 | 1074 | 17.3 | 25.5 | 44 | 38 | 1 | 1 | 999 | 8 | 5 |
| R210 | 05.01.23 | 0 | 1075 | 754 | 1164 | 858 | 16.7 | 17.8 | 53 | 51 | 1 | 1 | 999 | 9 | 4 |
| R219 | 05.09.23 | 1 | 411 | 539 | 533 | 575 | 25.4 | 25.2 | 19 | 18 | 4 | 0 | 3 | 5 | 0 |
| R221 | 05.10.23 | 1 | 493 | 470 | 613 | 588 | 27.6 | 28.8 | 25 | 21 | 6 | 0 | 7 | 7 | 0 |
| R227 | 05.23.23 | 0 | 1033 | 1098 | 1042 | 1220 | 20.0 | 21.2 | 41 | 44 | 2 | 1 | 999 | 9 | 6 |
| R228 | 05.23.23 | 1 | 514 | 400 | 645 | 499 | 26.9 | 27.3 | 31 | 29 | 4 | 0 | 5 | 7 | 6 |
| R233 | 05.31.23 | 0 | 1174 | 742 | 1206 | 803 | 22.2 | 21.0 | 47 | 50 | 3 | 1 | 999 | 11 | 5 |
| R237 | 06.06.23 | 1 | 789 | 1120 | 924 | 1233 | 23.8 | 22.7 | 27 | 31 | 4 | 1 | 999 | 7 | 0 |
| R248 | 07.11.23 | 1 | 494 | 572 | 620 | 695 | 26.3 | 28.7 | 43 | 36 | 5 | 0 | 3 | 6 | 6 |

The highest four CO_2_ levels (Aranet > 1300ppm) were recorded on trips in October 2022 (n=3) and January 2023 (n=1). On each of these trips, the relative humidity was 40-52%, the occupancy was medium to high, and all of the bus windows were closed. In contrast, on six trips in April (n=2), May (n=3), and July (n=1) 2023, even at high occupancy, low CO_2_ levels (Aranet < 700ppm) were recorded. For these trips, the relative humidity was 13-43%, and all of the buses had three to seven windows open.
